# Neural Correlates of Working Memory Deficits in Different Adult Outcomes of ADHD: An Event-Related Potential Study

**DOI:** 10.3389/fpsyt.2020.00348

**Published:** 2020-05-01

**Authors:** Xixi Zhao, Hui Li, Encong Wang, Xiangsheng Luo, Chuanliang Han, Qingjiu Cao, Lu Liu, Jin Chen, Changming Wang, Stuart J. Johnstone, Yufeng Wang, Li Sun

**Affiliations:** ^1^The National Clinical Research Center for Mental Disorders & Beijing Key Laboratory of Mental Disorders, Beijing Anding Hospital, Capital Medical University, Beijing, China; ^2^Advanced Innovation Center for Human Brain Protection, Capital Medical University, Beijing, China; ^3^Department of Child Psychiatry, Peking University Sixth Hospital/Institute of Mental Health, Beijing, China; ^4^National Clinical Research Center for Mental Disorder & Key Laboratory of Mental Health, Ministry of Health (Peking University), Beijing, China; ^5^Unit of Psychological Medicine, Beijing Chao-Yang Hospital, Capital Medical University, Beijing, China; ^6^State Key Laboratory of Cognitive Neuroscience and Learning & IDG/McGovern Institute for Brain Research, Beijing Normal University, Beijing, China; ^7^School of Psychology, Brain & Behaviour Research Institute, University of Wollongong, Wollongong, NSW, Australia

**Keywords:** attention deficit/hyperactivity disorder (ADHD), remitters, working memory (WM), n-back, event-related potentials (ERPs), P3

## Abstract

**Background:**

We investigated working memory (WM) processing in a longitudinal sample of young adults with persistent and remittent childhood-onset ADHD to investigate the neural correlates of working memory with adult outcomes of ADHD.

**Methods:**

Forty-seven young Chinese adults who had been diagnosed with ADHD during childhood underwent follow-up assessments for an average of 9 years. The ADHD sample consisted of 25 ADHD persisters (mean age =18.38 ± 0.5 years) and 22 remitters (mean age = 18.78 ± 1.10 years), who were compared with 25 sex ratio- and IQ-matched healthy adults (mean age = 19.60 ± 1.22 years) in a verbal n-back task.

**Results:**

No differences in behavioral measures were observed across the three groups. Compared with the healthy controls, the ADHD persisters and remitters had larger N1 amplitudes and smaller P2 amplitudes, while no significant differences between the persistence and remission groups were observed. The P3 amplitudes of the remission and control groups were higher than that of the persistence group, but there was no significant difference between the remitters and healthy controls.

**Conclusion:**

The P3 amplitudes reflecting postdecisional processing and/or WM updating were sensitive to ADHD remission, as they might improve concurrently with ADHD symptoms. These results indicate that the N1, P2, and P3 components of WM processing might be potential biomarkers for different ADHD outcomes.

## Introduction

Attention-deficit/hyperactivity disorder (ADHD), which occurs in children and adolescents, is now known to have a long-term impact throughout the lifespan of an individual (1). Although some young adults experience the remission of clinical symptoms and thus no longer meet the diagnostic criteria for ADHD, the persistence rate of ADHD is still high. A prospective 10-year follow-up study showed that 35% of participants continued to meet the full DSM-IV criteria for ADHD, 22% met the subthreshold criteria, 15% were functionally impaired, and 6% were receiving treatment although they did not meet the criteria for ADHD and were functioning well in their daily lives (2). Recent studies have suggested that remission arises due to compensatory neural reorganization and neurodevelopment (3–5). To determine the different outcomes of ADHD and analyze the correlations with clinical symptoms, event-related potential (ERP) indicators may be used as biomarkers to predict outcomes and thus better understand the mechanism of recovery. The identification of the characteristics that can be used to distinguish individuals with different outcomes may inform the development of novel treatment strategies that improve the outcomes in ADHD patients.

It has been proposed that the cognitive processes associated with the persistence of ADHD during the developmental stages may be separate from those linked to the remission of the disorder. However, the evidence remains controversial, as working memory (WM) is positively correlated with symptomatic improvement. Loo holds the view that the cognitive measures of WM can differ between ADHD remitters and persisters (6). Other researchers hold that working memory is not sensitive to ADHD persistence or remission, as ADHD remitters have shown an intermediate pattern between those of persisters and control participants, without significant differences from either group in a cued continuous performance task (7). As it is one of the main components of executive function, WM enables individuals to temporarily hold and manipulate information for a short period (a few seconds), helping them make decisions or generate responses based on that information (8). WM deficits are present in a substantial portion of patients with ADHD (9–14). A review of 13 studies reported that, compared with controls, adults with ADHD had impaired verbal WM according to their performance on the digit span task from the WEIS IQ test (15). Many researchers have found that adult ADHD still is associated with deficits in working memory, but the evidence for ADHD remission in WM is limited and inconclusive.

Neurophysiological studies have provided a deeper understanding of the neural substrates of WM in ADHD patients. An increasing number of studies have used ERPs to examine neural mechanisms underlying WM in ADHD patients, which have excellent temporal resolution and are useful to characterize the rapid recruitment of neural generators. Different sensory/cognitive processing stages involved in the task can generate various ERP components. Decreased N1, P2, and P3 amplitudes in adult ADHD have been documented during a classical n-back verbal WM task (13, 16). Sensory processing and the process of encoding in the early stages of the WM task are related to the N1 component, which usually corresponds to visual processing and the orienting of attention (17). Increased N1 amplitudes in individuals with ADHD may reflect additional neuronal activity associated with shifting one's attention during a selection task (18). The frontal P2 component reflects top-down matching between sensory inputs and stored memory traces (19, 20). Larger P2 amplitudes may indicate that excessive cognitive resources are allocated to the matching procedure in WM updating (21). During a visual oddball task, adults with ADHD exhibited large P2 amplitudes at the vertex, suggesting that atypical brain activity is associated with sensory processing in adults with ADHD (22). It is widely assumed that the P3 component, with a maximum peak amplitude between 300 and 500 ms post stimulus, is thought to reflect neural activity related to attention and working memory processes (23–26). According to the resource allocation theory of P3, an increased working memory load can decrease P3 amplitudes, as more attention resources are needed to maintain and update working memory (27). Some studies have shown that P3 amplitudes are sensitive to memory loads such that they decrease as *n* increases in n-back tasks (24).

ERPs have also been used to estimate the processes underlying the persistence and remission of ADHD. One longitudinal electrophysiological study used a cued continuous performance test (CPT) to investigate the course of multiple impaired cognitive brain functions from childhood to adulthood in individuals with ADHD. The authors reported that significant differences in Cue P3 and No-Go P3 between the ADHD group and typically developing controls became nonsignificant in early adulthood (28). In contrast, another study reported that the Cue P3 amplitude in ADHD remitters did not significantly differ from that in controls during a cued flanker CPT (7). A longitudinal study with 87 ADHD persisters, 23 remitters, and 169 controls was conducted to determine whether cognitive and neurophysiological impairments on a performance-monitoring task can be used to distinguish between ADHD persisters and remitters. The results showed that all cognitive performance and event-related potential measures were impaired in ADHD persisters compared with the controls. The ADHD remitters differed from the persisters and were indistinguishable from the controls in the number of congruent errors, reaction time variability, error-related negativity, and error-related positivity. However, the number of incongruent errors, mean reaction time, and N2 amplitude could not distinguish the remitters from the other groups (29). Although different measures were used, these electrophysiological findings demonstrated there are neural mechanisms underlying cognitive function in individuals with different ADHD outcomes. However, the neurophysiological characteristics of WM processing in ADHD remitters and persisters are still not clearly understood.

To illustrate the patterns of WM processing that are specific to different ADHD outcomes, we measured ERPs in young adults with different ADHD outcomes who completed follow-up assessments after an initial diagnosis during an n-back task. The present study hypothesized that working memory performance is poorer in ADHD persisters than in ADHD remitters and healthy controls. We predicted that the N1 and P2 components would show normalized patterns in ADHD remitters, as in the healthy controls, but show abnormalities in ADHD persisters compared with the healthy controls. We further predicted that ADHD persisters would show reduced P3 amplitudes compared with both the healthy adults and remitters and that the abnormal ERP amplitudes in the ADHD persisters would be correlated with ADHD symptom severity.

## Methods

### Sample

ADHD data were gathered from subjects in a previous study in which subjects were recruited from pediatric psychiatry clinics at Peking University Sixth Hospital from 1999 to 2009 and were contacted to participate in a follow-up interview after they were 18 years old (ranging from 18 to 24 years old) from 2007 to 2013 (30). The initial diagnosis of ADHD was based on the criteria of the Diagnostic and Statistical Manual of Mental Disorders, Fourth Edition (DSM-IV). The n-back task was completed by 47 young adults who were diagnosed with ADHD during childhood and underwent follow-up assessments for an average period of 9 years after the initial assessment. The ADHD study population consisted of 25 ADHD persisters (mean age =18.38 ± 0.5 years) and 22 remitters (mean age = 18.78 ± 1.10 years). We also included data from 25 sex ratio- and IQ-matched healthy adults (mean age = 19.60 ± 1.22 years) from our previous project as a control group (10, 31).

According to the childhood records, in the persistence group, 16 patients met the criteria for the predominantly inattentive subtype (ADHD-I), and nine met the criteria for the combined subtype (ADHD-C) of ADHD. In the remission group, 16 met the criteria for the ADHD-I subtype, and six met the criteria for the ADHD-C subtype of ADHD. At the follow-up assessment, four persisters who had been diagnosed with the ADHD-I subtype during childhood presented with ADHD-C, three persisters who formerly had ADHD-C had the hyperactive-impulsive subtype (ADHD-H), and five persisters who formerly had ADHD-C had ADHD-I. None of the patients received nonpharmacological treatment from childhood to adulthood. None of the participants received pharmaceutical treatments for ADHD in the month prior to the follow-up assessment. Other current psychopathologies were assessed *via* the Structured Clinical Interview for DSM-IV Axis I Disorders (SCID) during the follow-up interview. In an effort to recruit an ADHD sample that was representative of the clinical population, we did not exclude individuals with comorbidities. In the persistence group, there were seven participants with major depression disorder (MDD), one participant with obsessive–compulsive disorder (OCD), one participant with social phobia, and three participants with substance abuse. In the remission group, there were two participants with dysthymia.

The healthy participants in the control group were recruited from local universities or communities and interviewed to ensure an absence of past or current ADHD, autism, depression, anxiety, schizophrenia, bipolar disorder, or other mental disorders. All of the participants were screened for any potential comorbidities using the Structured Clinical Interview for DSM-IV Axis I Disorders by a qualiﬁed psychiatrist. We used the following exclusion criteria for ADHD patients and healthy controls: (1) major neurological dysfunction and psychosis, (2) pervasive developmental disorders, (3) score of <80 on the Chinese version of the revised full Wechsler Adult Intelligence Scale (WAISRC), and (4) non-right handedness. The studies involving human participants were reviewed and approved by the Research Ethics Review Board of XX University Institute of Mental Health. The patients/participants provided their written informed consent to participate in this study. The participant demographics are presented in **Table 1**.

**Table 1:** Demographic and clinical measures for ADHD persistence group, remission group, and healthy control group.

Items	ADHD Persistence *n*=25	Remission *n*=22	Control *n*=25	*F/*χ^2^	*p*
**Sex**	M 20; F 5	M 19; F 3	M 19; F 6	0.83	0.703
**Age (years, mean ± SD)**	18.38 ± 0.50	18.76 ± 1.10	19.60 ± 1.22	9.99	<0.001
**IQ (mean ± SD)**	110 ± 8.679	113 ± 2	115 ± 7.918	1.79	0.174
**Childhood ADHD type**	16 I, 9 C	16 I, 6 C	n/a		
**Adult ADHD type**	17 I, 3 H, 5 C	n/a	n/a		
**Current ADHD symptoms (mean ± SD)**					
***Inattentive***	6.80 ± 1.71	2.00 ± 1.11	1.001.04	134.51	<0.001
***Hyperactivity-impulsive***	4.00 ± 2.57	1.09 ± 1.11	0.40 ± 0.65	32.18	<0.001
***Total***	10.80 ± 2.71	3.09 ± 1.82	1.40 ± 1.23	152.15	<0.001
***Comorbidities***	7 MDD, 1 OCD, 1 Social phobia, 3 Substance abuse	2 Dysthymia	n/a		

IQ, estimated full IQ of WAISRC; ADHD, attention-deficit/hyperactivity disorder; MDD, major depression disorder; OCD, obsessive-compulsive disorder; I, inattentive subtype; H, hyperactive-impulsive subtype; C, combined subtype; χ^2^, chi-square test.

### Measures

#### ADHD Diagnosis

Childhood ADHD was diagnosed according to the DSM-IV criteria with a semistructured interview and the Clinical Diagnostic Interview Scale (CDIS). Our group previously translated the Chinese version of the CDIS (32). The test has good sensitivity (97.2%) and specificity (100%) for ADHD.

Adulthood ADHD was diagnosed during the follow-up *via* the Chinese version of the Conner's Adult ADHD Diagnostic Interview (CAADI). It comprises questions on 18 symptoms of ADHD, demographic characteristics, the developmental course, ADHD risk factors, and comorbidities based on the DSM-IV diagnostic criteria. The Kappa coefficient was 0.67 in an American population and 1.0 in our study population (33). The interviewers were trained and experienced psychiatrists who were not blinded to the baseline information. For the 47 participants, we conducted follow-up interviews with the young adults alone (N = 16, 34.04%) or with their parents (N = 31, 65.96%). When both the parents and young adults were interviewed, a symptom was considered present if either the parents or participants supported it.

In our cohort, the following groups were defined: (a) participants meeting the full DSM-IV criteria for ADHD (“syndromatic persistence”), (b) participants meeting the subthreshold DSM-IV criteria (i.e. more than half of the symptoms required for a full diagnosis, “symptomatic persistence”), (c) participants not meeting the criteria for (a) or (b) who were functionally impaired with a DSM-IV Global Assessment of Functioning (GAF) score of ≤60 (“functional persistence”), and (d) participants not meeting the criteria for (a), (b), or (c), thus exhibiting full remission. In the current study, all individuals in the ADHD persistence group met the criteria for “syndromatic persistence,” and all of the participants in the remission group met the criteria for full remission.

#### Working Memory Task: N-Back

**Figure 1:**
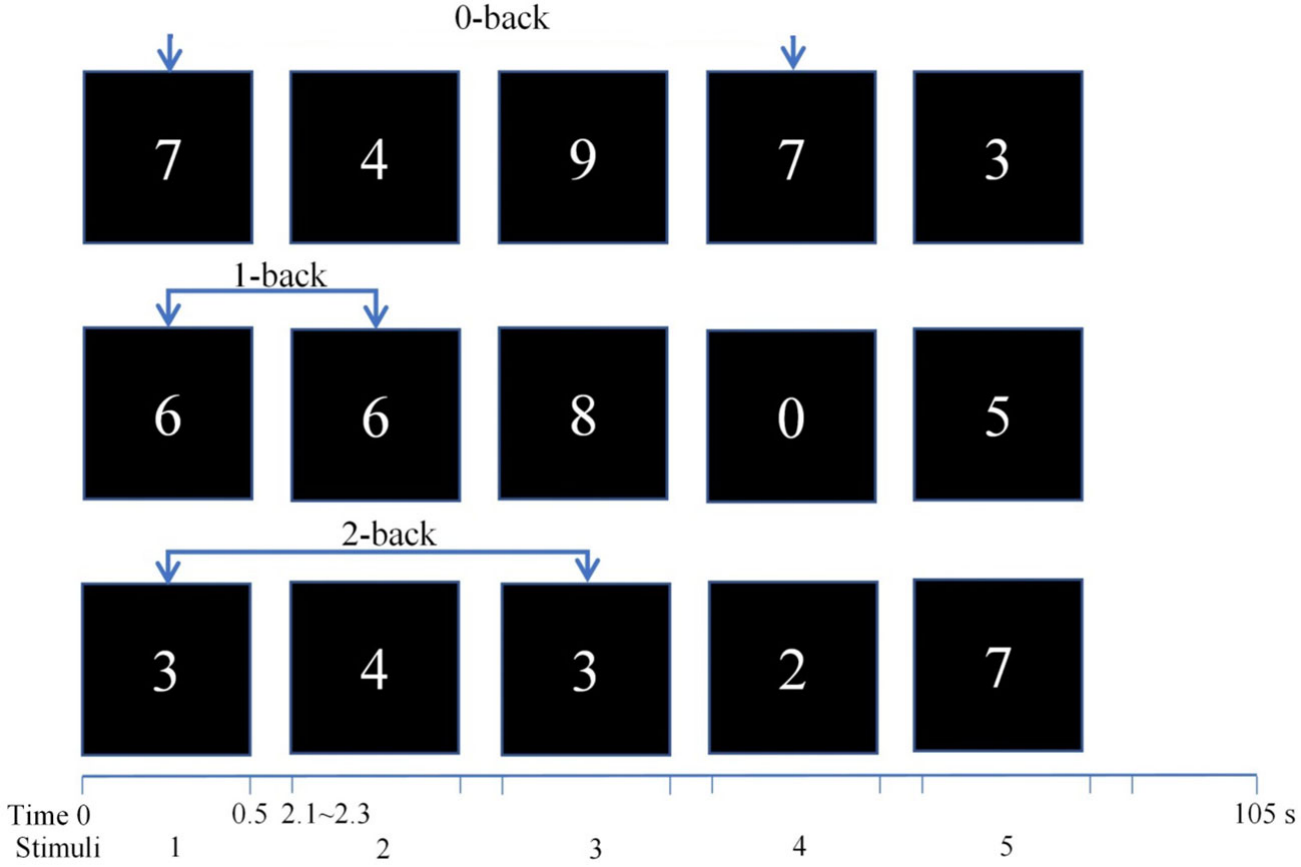
n-back task with three loads: 0-back, 1-back, and 2-back. A response was required whenever the current stimulus matched the number 7 and the stimulus one or two positions back in the sequence.

Working memory was tested using an n-back task presented by the software package E-Prime (Psychology Software Tools, Pittsburgh, PA) (34). We manipulated the memory load (0-, 1-, 2-back) and stimulus type (nontarget, target), which were independent variables within participants. For the 0-back load, the target was the number “7.” For the 1-back and 2-back loads, the participants were asked to identify the current number (from 0 to 9) that was shown for 500 ms and to recall at the same time the number(s) that was shown in the previous one trial (1-back) or two trials (2-back). For example, the second 6 in the sequence 6-6-8-0-5 was a target in the 1-back task. In particular, the second and third 6s in the sequence 6-6-6-0-5 were targets in the 1-back task. In addition, the second 3 in the sequence 3-4-3-2-7 was a target in the 2-back task. In particular, the second 3 and 4 in the sequence 3-4-3-4-7 were both targets in the 2-back task. Before each block, the participants were shown which working memory load condition to expect for exactly 1,500 ms. Stimuli consisted of a continuous stream of numbers that appeared in the center of the screen and were presented for 500 ms with an interstimulus interval that varied randomly between 1,600 and 1,800 ms. A total of 12 task blocks (540 trials) were presented, and each block consisted of three load conditions (0-back, 1-back, and 2-back), with 15 trials for each load. In all conditions, the targets occurred randomly in 33% of the trials, which means that there were 60 trials that were considered targets for each load. The participants had a short break between blocks. The total duration of the experiment was approximately 30 min (see **Figure 1**).

The participants were seated in a dimly lit and sound-attenuated room. We instructed participants to respond as quickly and accurately as possible whether each number matched the target. They used the index finger of their right hand to press a button indicating the target was present and the middle finger of their right hand to press a different button indicating a non-target was present. They were required to respond as accurately and quickly as possible. All participants performed a practice block of 45 trials before the beginning of the first block of experimental trials to ensure they understood the task.

#### Electrophysiological Recording and Processing

The participants were seated comfortably in a dimly lit room with a low level of environmental noise. They were instructed to remain relaxed and still. We recorded EEG signals using a 128-channel system (HydroCel Geodesic Sensor Net, Electrical Geodesics, Inc., Eugene, OR) with a 1,000-Hz sampling rate, the impedance values were less than 50 kΩ, and the Cz electrode was used as the reference electrode. The data were analyzed offline with EEGlab. The raw EEG recordings were downsampled to 250 Hz and re-referenced to the average amplitude of the signals from all electrodes. A bandpass filter was applied from 0.05 to 30 Hz. All trials were visually inspected for electrical or obvious movement artifacts, and sections of data containing artifacts were removed manually. Then, we used independent component analysis (ICA) to decompose the measured EEG signals into independent components, and the components sensitive to eye blinks, eye movements, head movements, heartbeats, and other visually identified artifacts were removed (35).

### Data Analysis

#### Behavioral Data

The outcome measures included the response time (RT), performance accuracy, and intraindividual standard deviation (ISD). These outcomes were calculated separately for the 0-back, 1-back, and 2-back load tasks. Responses faster than 200 ms and slower than 2,000 ms were considered incorrect. All of the ADHD persisters, remitters, and healthy control participants were included in the behavioral and ERP analysis.

#### ERP Data

The ERP data were processed using EEGlab. Only correct trials were included for further analysis. Artifacts were removed by removing segments with an absolute difference larger than 200 mV or a voltage step per sampling point larger than 50 mV. Baseline correction was applied from −200 ms until stimulus onset. Epochs were averaged from 200 ms before stimulus onset to 1,000 ms poststimulus onset individually for the stimuli in each load condition. ERP sites were selected based on the reported references (26, 36, 37) and brain electrical activity mapping of the three groups (**Supplemental Figures S1** and **S2**). As the early ERP components were the largest at the anterior sites, we performed N1 and P2 quantification on individual ERP data at Fz in windows spanning 40–120 ms (N1) and 120–220 ms (P2) poststimulus. As the P3 amplitude was the largest at the parietal sites, we selected Pz for quantification in a window spanning 200–500 ms poststimulus. We also analyzed the ERPs using cluster electrodes to test the replicability of the results. N1 and P2 were investigated at the averaged F1, F2, and Fz, and P3 was investigated at the averaged P1, P2, and Pz (for more details, see the **Supplemental Materials**). We calculated the average amplitude in a symmetric 50 ms interval around the peak latency. The latency of each ERP component was measured from stimulus onset to the time of the maximum value, and we measured the amplitude from the prestimulus baseline to the maximum value.

### Statistical Analyses

All analyses were performed in SPSS version 18. We used analyses of variance (ANOVAs) to assess the differences in the demographic variables among the three groups. For behavioral measures and ERP data, we used 3*3 ANOVAs with Group (remission, persistence, healthy control) and Load (0-back, 1-back, 2-back) as the main factors, and we included Age as a covariate. We used similar ANOVAs to assess the behavioral performance measures (RT, accuracy, and ISD) and ERP components (N1, P2, and P3). Furthermore, we used Pearson's correlation analyses to examine the relationships between ADHD symptom scores/behavioral performance and ERP components that had significant group differences. Statistical significance was set to be α < 0.05, and the Bonferroni correction was applied. To remove the effect of comorbidities and covariates, we repeated the statistical analysis without including data from patients with comorbidities (for more details, see the **Supplemental Materials**).

## Results

### Demographic and Clinical Measures

We found no significant differences across the three groups in terms of the sex ratio or estimated IQ. The mean age of the healthy controls was higher than that of the ADHD persistence and remission groups, and there was no significant difference in age between the ADHD persistence and remission groups. According to the clinical measures, individuals in the ADHD persistence group had significantly higher scores for inattentive and hyperactive-impulsive symptoms compared with those in the remission group and healthy controls (see **Table 1**).

### Behavioral Results of the N-Back Task

**Figure 2:**
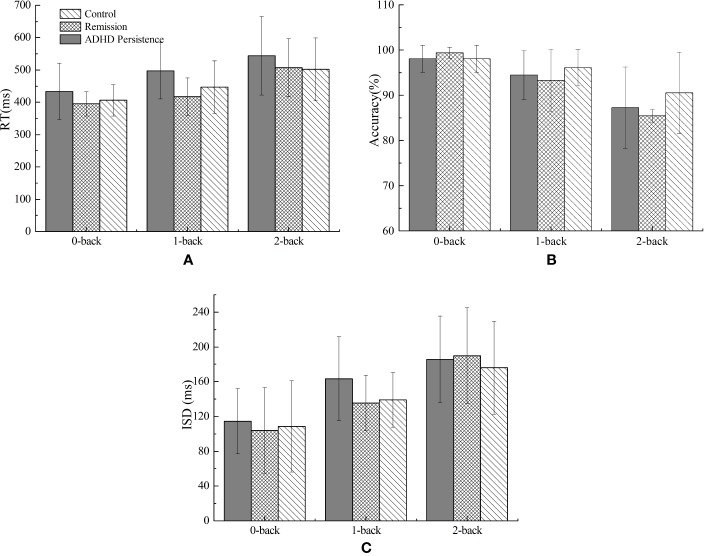
Behavioral results of the n-back task for ADHD persistence group, remission group, and control group. **(A)** Response time (RT, ms), **(B)** Accuracy (%), **(C)** Intra-individual standard deviation (ISD, ms). The error bars stand for standard deviations.

In terms of the mean correct RT, mean accuracy, and mean ISD in each memory load condition (0-, 1-, and 2-back) for the three groups, we only found main effects of Load. As the memory load increased, RT increased [F (1.53, 105.86) = 86.42, P < 0.0001, η_p_² = 0.556], accuracy decreased [F (1.40, 96.78) = 58.17, P < 0.001, η_p_² = 0.457], and ISD increased [F (2, 13) = 80.22, P < 0.001, η_p_² = 0.538]. There were no significant Load * Group interactions or main effects of Group (**Figure 2**).

### ERP Results

**Figure 3:**
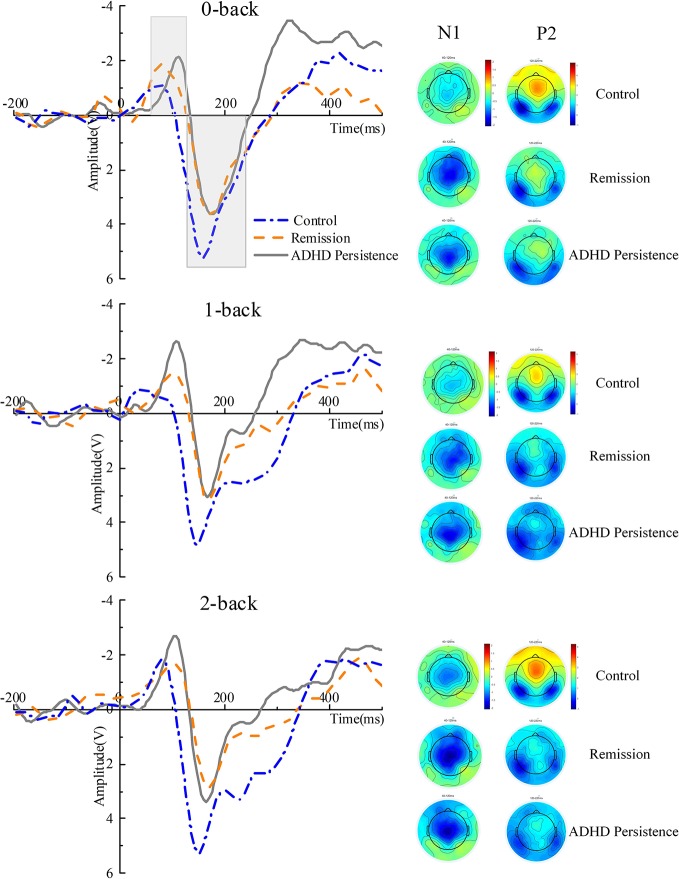
ERP waveforms at Fz and topographic maps. The N1 and P2 were quantified as the maximum negatively between 40 and 120 ms and positively between 120 and 220 ms, respectively, after stimulus-onset at electrode site Fz during the n-back task. The time window of the topographic map is the same as the N1 and P2, respectively.

**Figure 4:**
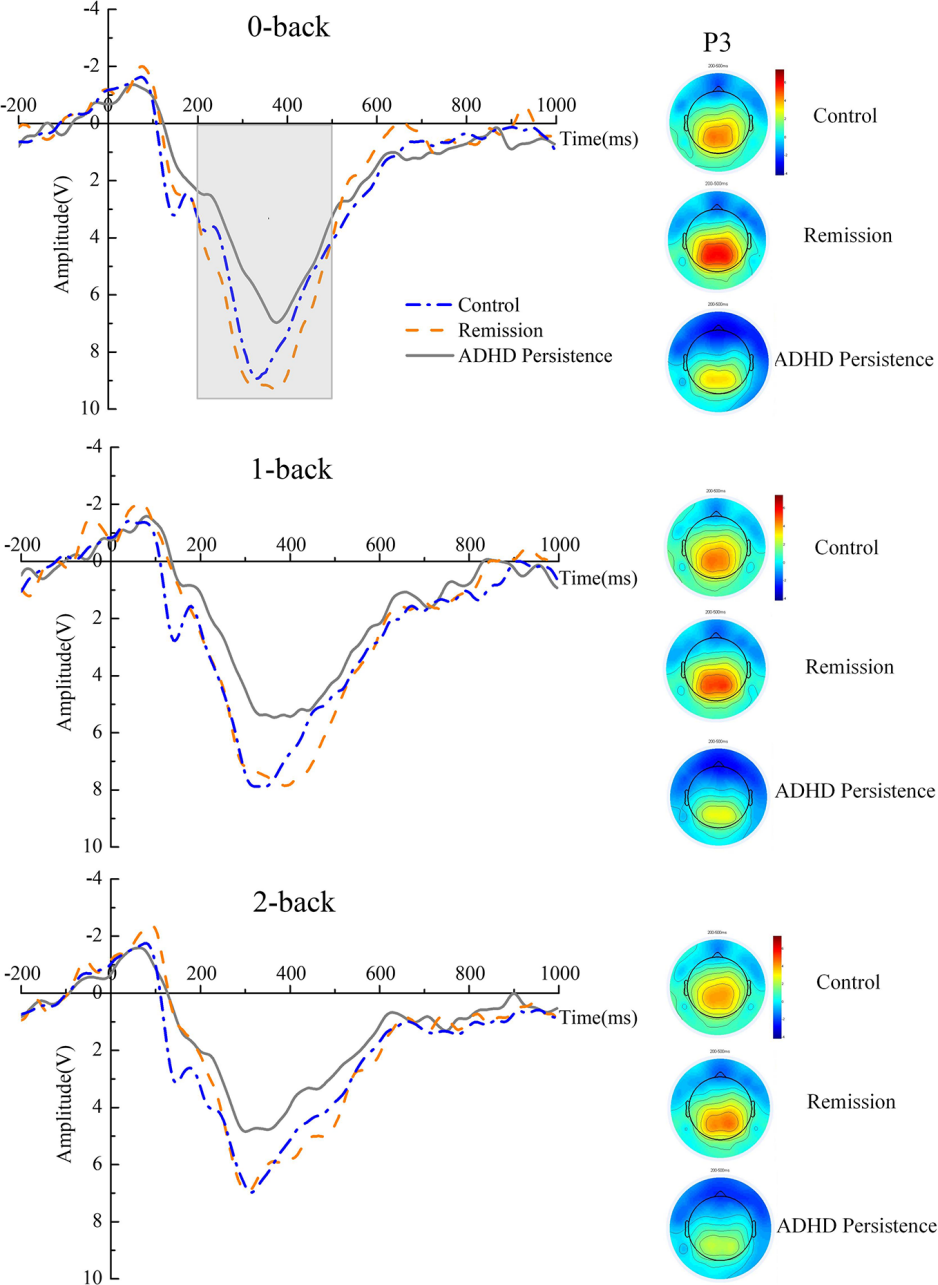
ERP waveforms at Pz and topographic maps. The P3 component was quantified as the maximum positivity between 200 and 500 ms after stimulus-onset for each task for electrode site Pz during the n-back task. The time window of the topographic map is the same as the P3.

Grand-mean waveforms for the n-back task for each WM load condition for the three groups are shown in **Figure 3** (Fz) and **Figure 4** (Pz). For the N1 component, we found a significant main effect of Group on peak amplitude [F (2, 68) = 4.302, P = 0.017, η_p_² = 0.112] and peak latency [F (2, 68) = 6.249, P = 0.003, η_p_² = 0.155]. An additional LSD analysis showed that the N1 amplitude was lower in healthy controls than in the persistence (P = 0.025) and remission groups (P = 0.041), although there was no significant difference between the persistence and remission groups (P = 0.717). N1 latency showed a similar pattern, with a shorter latency in the healthy controls than in the persistence (P = 0.001) and remission groups (P = 0.027) and no significant difference between the persistence and remission groups (P = 0.158). There were no significant interactions or other main effects.

For the P2 amplitude, we only found a main effect of Group [F (2, 68) = 5.655, P = 0.005, η_p_² = 0.143]. The P2 amplitude in the healthy controls was higher than that in the persistence (P = 0.003) and remission groups (P = 0.007), but there was no significant difference between the persistence and remission groups (P = 0.634). There were no significant interactions or main effects on P2 peak latency.

The P3 amplitude decreased as the memory load increased [F (2, 136) = 27.49, P < 0.001, ηp² = 0.285]. No factors interacted with Group, suggesting that the three groups exhibited the same trend across the different load conditions. There was a significant main effect of Group [F (2, 68) = 3.214, P = 0.046, ηp² = 0.086], with an additional analysis indicating that the P3 amplitude in healthy controls was larger than that in the persistent group (P = 0.025) and that the P3 amplitude in the remission group was larger than that in the persistent group (P = 0.041). However, there was no significant difference in P3 amplitude between the healthy controls and remission group (P = 0.717). We conducted a parallel analysis for P3 peak latency and found no significant interactions or main effects (for more details see **Supplemental Tables 1** and **2**).

### Correlations Between Behavioral Performance and ERPs

In the control group, the P3 amplitude was negatively correlated with accuracy in the n-back task (0-back: r = −0.524, p = 0.063; 1-back: r = −0.565, p = 0.027; 2-back: r = −0.563, p = 0.027). We did not detect this correlation in the ADHD persistence or remission groups. No significant correlations were found with RT or ISD.

### Correlations Between ADHD Symptoms and ERPs

**Figure 5:**
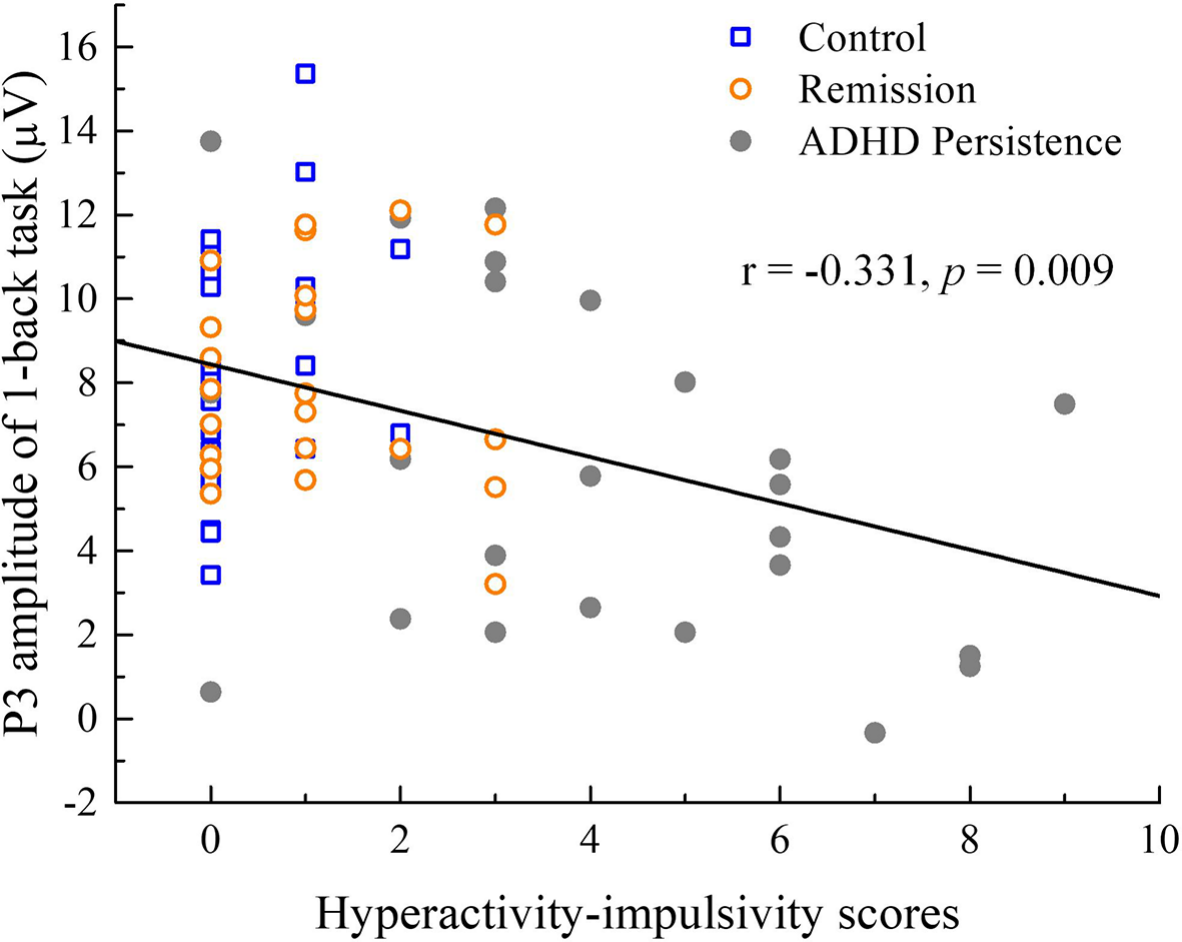
Correlations between ADHD symptoms and ERP measures. In all participants, the hyperactivity-impulsivity scores had a negative correlation with the P3 amplitude of 1-back.

In all participants, the hyperactivity-impulsivity scores were negatively correlated with the P3 amplitudes observed during the 1-back task (r = −0.331, P = 0.009). In the ADHD persistence group, the hyperactivity-impulsivity scores were negatively correlated with the P3 amplitudes observed during the 1-back task (r = −0.405, P = 0.027), while there were no significant correlations in the remission group or healthy control group (**Figure 5**).

## Discussion

In this study, we investigated the electrophysiological indices of working memory with respect to different ADHD outcomes. The ADHD persistence and remission groups showed comparable behavioral performance during the n-back task. We found that the ADHD remission group had improved neurophysiological WM processing performance (P3) compared with the persistence group. Conversely, the neurophysiological measures of N1 and P2 did not distinguish the remission group from the persistence group, although these components did significantly differ between the ADHD persistence group and healthy controls.

As expected, the behavioral and neurophysiological results showed the main effects of task load. We found that RT increased, accuracy decreased, and ISD increased with increasing task difficulty in all groups. Furthermore, all three groups exhibited a reduced P3 amplitude during the 1-back and 2-back load tasks compared with the 0-back load task. According to the resource allocation theory of P3 (38), a typical decrease in P3 amplitude with an increase in WM load is related to the allocation of more attentional resources to the WM-updating subtask (26). Interestingly, although the P3 amplitude was negatively correlated with the accuracy of performance in the n-back task in the control group, this correlation was absent in the ADHD persistence and remission groups. This result might reflect two competing processes; specifically, P3 may decrease due to the allocation of resources to the “subtask” in healthy controls, while ADHD patients may have a lower capacity or inefficient resource allocation, resulting in reduced P3 amplitudes regardless of the task difficulty. Consistent with our hypothesis, the P3 amplitude was significantly different in the ADHD persistence group vs. the healthy control group, whereas there were no significant differences between the ADHD remission group and healthy control group. This result supports the idea that the parietal P3 component is related to the allocation of attentional resources for updating working memory information (25). Similarly, Kim found that the P3 amplitude was smaller in an ADHD group compared with controls at parietal–occipital sites, suggesting ineffective allocation of the attentional resources involved in encoding information in WM (12). Importantly, we found that the P3 peak amplitude was negatively correlated with the hyperactivity-impulsivity scores. The objective neurobiological characteristics might be important in understanding the disease mechanisms and developing individual interventions. This finding indicates that the inefficient allocation of attentional resources might be a component of the neural mechanisms underlying hyperactivity-impulsivity symptoms. Researchers have found evidence that neurofeedback (NF) is effective in treating adult ADHD in the long term (39). This finding also indicates that P3 can be used as an evaluation index in NF training.

Inconsistent with our expectations, the early ERP components generated at the prefrontal region were abnormal in the remission group. We found that the N1 and P2 peak amplitudes in the ADHD persistence and remission groups were significantly different from those in the healthy control group. N1 relates to the early stages of sensory processing and is implicated in the mechanism of encoding information in WM. Increased N1 amplitudes in patients with ADHD suggest the presence of additional neuronal activity associated with shifting one's attention. One study found that the medial frontal N1 amplitude was significantly higher in adults with ADHD than in healthy controls during a Go/No-go task. Given this finding and their finding that the P3 amplitude is reduced in adults with ADHD, the authors suggested that adults with ADHD might engage higher levels of attention compared with healthy adults to compensate for their impairment and achieve the same performance (18). Consistent with this suggestion, we found a higher N1 amplitude in the ADHD persistence and remission groups, perhaps indicating that ADHD patients suffer from encoding deficiencies and require more neural activity to direct attention to stimuli. Moreover, our data indicate that the ADHD remission group may have had this encoding impairment despite exhibiting the remission of symptoms. Furthermore, the P3 in the remission group was similar to that in the persistence group despite the N1 being larger in the remission group than in the healthy controls, which might reflect important heterogeneity in the ADHD population. Indeed, P3 and N1 recorded during the n-back task may be useful neurobiological markers of the recovery trajectory in individuals with ADHD.

We found that individuals in the ADHD persistence and remission groups showed a reduced P2 peak amplitude compared with the healthy controls. The P2 component is thought to represent early encoding and retrieval phases of WM processing, following purely sensory-driven processes. In line with our findings, a previous study observed a reduced P2 amplitude in ADHD patients compared with controls, suggesting that the cortical generators involved in attention processing may be affected during the n-back task in individuals with ADHD (13). The fact that we observed a similar increase in frontal N1 and P2 amplitude in ADHD remitters and persisters indicates that the early processes of WM might not be closely involved in the neuro-mechanisms of different ADHD outcomes. Furthermore, impaired frontal N1 and P2 activity during WM processes might be an endo-phenotype of ADHD and quantitative indices of disease liability or risk.

A recent review demonstrated that individuals with remittent ADHD have some neural features indistinguishable from those of controls, while some deep brain anomalies may persist even during remission (3). A previous study also revealed that individuals with remittent ADHD differed from those with persistent ADHD in measures related to attention-vigilance and neurophysiological error processes. However, cognitive measures of executive control, speed of processing, and conflict monitoring did not distinguish the individuals with remittent and persistent ADHD (29). Our results were also consistent with this pattern. P3 during WM processing in individuals with remittent ADHD was indistinguishable from that in controls, but N1 and P2 in individuals with remittent ADHD were abnormal compared to those in healthy controls, suggesting that different stages of WM processing might be involved in different mechanisms of ADHD.

## Limitations

Our data should be considered in light of several limitations. First, as we did not exclude participants with comorbidities, study population characteristics, such as the percentage of participants with depression, may have influenced the magnitude of the group differences. However, we included individuals with comorbidities to improve the representativeness of our study population, which may increase the generalizability of our data to individuals with a wide range of clinical conditions. When we repeated the analysis without the patients with comorbidities, the results showed similar tendencies. Second, the mean age of the control group was not matched to those of the ADHD persistence or remission groups. To address this issue, we used age as a covariate in the statistical analysis and adjusted our results accordingly. Finally, our sample size was too small to analyze the differences across ADHD subtypes. As this study is a cross-sectional study, the neurophysiological results cannot indicate the trajectories to ADHD remission and persistence. Additional longitudinal studies with larger sample sizes are needed to explore the changes in specific neurophysiological characteristics related to ADHD outcomes to provide more evidence of the mechanisms underlying remission.

## Conclusion

Overall, our results suggest that the encoding process during WM activity is impaired in both young adults with persistent and remittent ADHD. However, postdecisional processing and/or working memory updating reflected by P3 in the ADHD remission group was higher in those with fewer hyperactivity-impulsivity symptoms. Thus, the P3 component may be related to compensatory mechanisms in individuals who experience ADHD remission. These processes may be targets for nonpharmacological interventions or behavioral training, such as NF training, aimed at alleviating some of the long-term outcomes of ADHD. Additional studies should be conducted to investigate the neural sources and neurobiological mechanisms underlying markers of remission, with the goal of developing new interventions aimed at stimulating processes that are sensitive to remission and reducing the severity of the long-term outcomes of ADHD.

## Data Availability Statement

The datasets generated for this study are available on request to the corresponding author.

## Ethics Statement

This study was approved by the Research Ethics Review Board of Peking University Institute of Mental Health (No. 2013-35), and all participants provided informed written consent prior to the start of the study. In the first recruitment, the participants were children and the consents were obtained from their parents. In the second recruitment (follow-up), when the participants were in their adulthood, the consents were obtained from the participants themselves.

## Author Contributions

LS, HL, and XZ contributed to the conception of the study. LS, HL, and XZ contributed significantly to analysis and manuscript preparation. EW, XL, CH, QC, LL, and JC performed the data analyses and wrote the manuscript. CW, SJ, and YW helped perform the analysis with constructive discussions. All authors agree to be accountable for the content of the work.

## Funding

The study was funded by a grant from National Key Technology R&D Program (Qingjiu Cao, 2016YFC1306103) and was partially supported by the National Natural Sciences Foundation of China (LS, 81771479, 81971284), the Beijing municipal science and technology Program (LS,Z171100001017089, Z171100000117004), and research plan for innovation in clinical technology by Beijng Hospitals Authority (XMLX201805).

## Conflict of Interest

The authors declare that the research was conducted in the absence of any commercial or financial relationships that could be construed as a potential conflict of interest.
