# Supplementary material for: Neural Correlates of Working Memory Deficits in Different Adult Outcomes of ADHD: An Event-Related Potential Study
Source: Front Psychiatry. 2020 May 1;11:348. doi: 10.3389/fpsyt.2020.00348 (PMC7206828; doi:10.3389/fpsyt.2020.00348)
Supplement: Supplementary file 1 [file DataSheet_1.docx]

***Supplemental Materials***

**1. ERPs measures of clusters electrodes**

The amplitude and latency of N1 and P2 were measured at the cluster electrodes of F1, F2, and Fz. For P3 component, the cluster electrodes of P1, P2, and Pz were elected for quantification.

For N1 component, we found a main effect of Group for peak amplitude [F (2, 61.50) = 6.458, P = 0.003, η_p_² = 0.158] and peak latency [F (2, 68) = 4.339, P = 0.017, η_p_² = 0.112]. Further Bonferroni analysis showed the N1 amplitude in healthy controls were lower than that in the persistence (P < 0.001) and remission groups (P < 0.001), but we found no significant difference between the persistence and remission groups.

For P2 amplitude, we found a main effect of Load [F (2, 138) = 5.19, P = 0.007, η_p_² = 0.07] and a main effect of Group [F (2, 68) = 4.443, P = 0.015, η_p_² = 0.114]. The P2 amplitude in healthy controls were higher than those in the persistence (P = 0.032) and remission groups (P = 0.044), and we found no significant difference between the persistence and remission groups (P = 1.000). There were no significant interactions or main effects for the P2 peak latency.

For P3 amplitude, the main effect of Group showed a strong tendency towards statistical significance [F (2, 68) = 1.84, P = 0.051, η_p_² = 0.051]. We found no significant interactions or main effects for the P3 latency.

1. **Statistical analysis removing the effect of comorbidities**

Behavioral results of the n-back task

In terms of correct RT, mean accuracy, and mean ISD in each memory load condition (0-, 1-, and 2-back) for the three groups, only main effects of Load were found. RT increased with increasing memory load [F (1.46, 83.70) = 76.44, P < 0.001]. Accuracy decreased with increasing memory load [F (1.42, 81.06) = 62.58, P < 0.001]. ISD increased with increasing memory load [F (2, 114) = 62.48, P < 0.001].

ERP results

For N1 component, we found no significant interactions or main effects for the peak amplitude and latency. For P2 amplitude, only a main effect of Load was found [F (2, 66) = 15.13, P < 0.001]. We found no significant main effect of group [F (2, 65) = 2.55, P = 0.087]. There were no significant interactions or main effects of the P2 peak latency. P3 amplitude reduced as memory load increased [F (2, 108) = 22.104, P < 0.001]. The main effect of Group showed borderline level of statistical significance [F (2, 84) = 3.096, P = 0.053]. A parallel analysis was conducted for the P3 peak latency; there were no significant interactions or main effects.

1. **Statistical analysis removing the effect of covariates**

After removing the covariate, the statistical value is partially changed, but the experimental results did not change much.

For the N1 component, there was a significant main effect of Group for (1) peak amplitudes: without age as covariate [F (2, 69) = 4.525, P = 0.014, ηp² = 0.116] vs. with age as covariate [F (2, 68) = 4.302, P = 0.017, ηp² = 0.112]; (2) Peak latency: without age as covariate [F (2, 69) = 6.341, P = 0.0003, ηp² = 0.155] vs. with age as covariate [F (2, 68) = 6.249, P = 0.003, ηp² = 0.155].

For P2 amplitudes, only a main effect of Group was found: without age as covariate [F (2, 69) = 5.050, P = 0.009, ηp² = 0.128] vs. with age as covariate [F (2, 68) = 5.655, P = 0.005, ηp² = 0.143].

For P3 amplitudes, there was a significant main effect of Group for peak amplitudes: without age as covariate [F (2, 69) = 53.702, P = 0.030, ηp² = 0.097] vs. with age as covariate [F (2, 68) = 3.214, P = 0.046, ηp² = 0.086].

**Supplemental Table**

Table 1. Mean amplitude and latency of N1/P2/P3 in n-back task for three populations (mean±SD).

| Items | Task | Control（n=25） | ADHD  Persistence（n=25） | ADHD  Remission（n=22） |
| --- | --- | --- | --- | --- |
| N1 amplitude (μV) | 0-back | 2.34±2.30 | 0.80±1.41 | 1.21±2.48 |
|  | 1-back | 2.12±2.89 | 0.71±1.20 | 1.27±1.88 |
|  | 2-back | 2.49±2.81 | 0.70±1.86 | 1.18±1.73 |
| N1 latency (ms) | 0-back | 96.96±30.99 | 77.6±29.1 | 79.82±35.45 |
|  | 1-back | 94.88±30.08 | 72.16±28.69 | 76.91±33.24 |
|  | 2-back | 102.88±31.55 | 68.8±31.77 | 91.09±31.97 |
| P2 amplitude (μV) | 0-back | 6.08±2.13 | 4.56±2.88 | 5.08±3.49 |
|  | 1-back | 5.40±2.80 | 3.92±2.73 | 4.60±3.07 |
|  | 2-back | 6.44±2.65 | 4.21±2.96 | 4.24±3.24 |
| P2 latency (ms) | 0-back | 176.16±24.54 | 179.52±26.84 | 177.09±30.59 |
|  | 1-back | 164.32±25.69 | 177.6±24 | 181.09±27.66 |
|  | 2-back | 178.88±31.63 | 176.32±24.52 | 183.46±26.88 |
| P3 amplitude (μV) | 0-back | 9.13±4.39 | 7.23±3.73 | 9.20±3.02 |
|  | 1-back | 8.27±2.91 | 5.99±4.05 | 8.06±2.52 |
|  | 2-back | 7.69±3.42 | 5.02±3.47 | 6.87±2.95 |
| P3 latency (ms) | 0-back | 335.20±54.12 | 328.48±71.78 | 360.91±41.35 |
|  | 1-back | 328.48±61.26 | 346.56±57.40 | 360.00±54.47 |
|  | 2-back | 333.60±80.95 | 313.44±51.00 | 338.91±60.25 |

Table 2. Max and min amplitude and latency of N1/P2/P3 in n-back task for three groups.

| Items | | Task | Control（n=25） | ADHD  Persistence（n=25） | ADHD  Remission（n=22） |
| --- | --- | --- | --- | --- | --- |
| N1 amplitude (μV) | max | 0-back | 6.69 | 4.32 | 8.57 |
|  |  | 1-back | 9.61 | 3.06 | 4.91 |
|  |  | 2-back | 9.15 | 5.26 | 6.16 |
|  | min | 0-back | -1.40 | -1.00 | -0.81 |
|  |  | 1-back | -2.18 | -2.27 | -3.73 |
|  |  | 2-back | -1.85 | -2.16 | -1.02 |
| N1 latency (ms) | max | 0-back | 120.0 | 120.0 | 120.0 |
|  |  | 1-back | 120.0 | 120.0 | 120.0 |
|  |  | 2-back | 120.0 | 120.0 | 120.0 |
|  | min | 0-back | 44.0 | 44.0 | 44.0 |
|  |  | 1-back | 44.0 | 44.0 | 44.0 |
|  |  | 2-back | 44.0 | 44.0 | 44.0 |
| P2 amplitude (μV) | max | 0-back | 12.33 | 11.76 | 12.88 |
|  |  | 1-back | 9.82 | 8.73 | 8.18 |
|  |  | 2-back | 11.52 | 10.57 | 11.63 |
|  | min | 0-back | 2.48 | -1.46 | -0.38 |
|  |  | 1-back | 2.16 | -1.00 | -0.25 |
|  |  | 2-back | 2.04 | -1.22 | -1.99 |
| P2 latency (ms) | max | 0-back | 220.0 | 220.0 | 220.0 |
|  |  | 1-back | 220.0 | 220.0 | 220.0 |
|  |  | 2-back | 220.0 | 220.0 | 220.0 |
|  | min | 0-back | 136.0 | 144.0 | 124.0 |
|  |  | 1-back | 124.0 | 148.0 | 124.0 |
|  |  | 2-back | 140.0 | 136.0 | 124.0 |
| P3 amplitude (μV) | max | 0-back | 18.07 | 15.22 | 11.74 |
|  |  | 1-back | 15.37 | 13.75 | 12.10 |
|  |  | 2-back | 19.27 | 14.06 | 15.03 |
|  | min | 0-back | 1.12 | 0.64 | 2.26 |
|  |  | 1-back | 3.42 | 0.33 | 3.21 |
|  |  | 2-back | 3.27 | 0.95 | 2.95 |
| P3 latency (ms) | max | 0-back | 468 | 404 | 456 |
|  |  | 1-back | 476 | 404 | 460 |
|  |  | 2-back | 484 | 392 | 484 |
|  | min | 0-back | 212 | 204 | 304 |
|  |  | 1-back | 224 | 212 | 284 |
|  |  | 2-back | 224 | 204 | 260 |

**Supplemental Figure**

ADHD persisters

Healthy controls

2-back

1-back

ADHD remitters

0-back


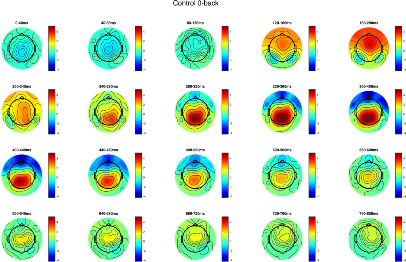

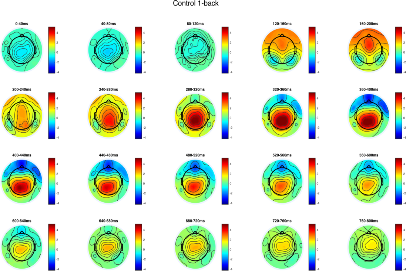

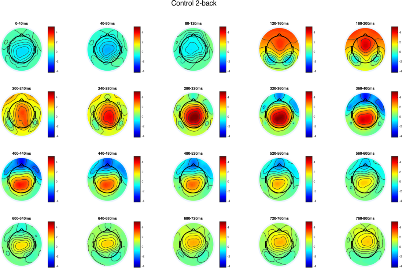

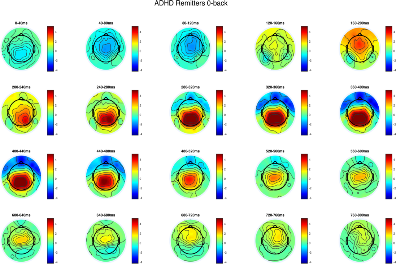

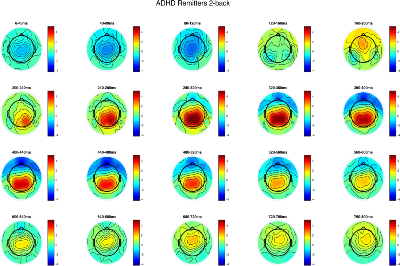

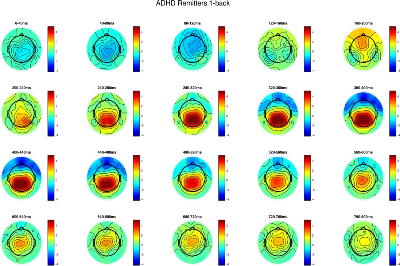

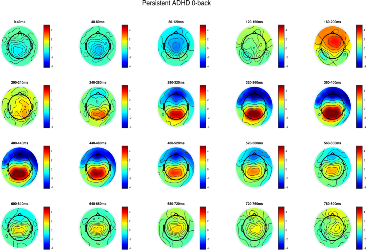

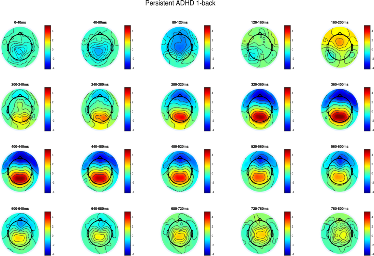

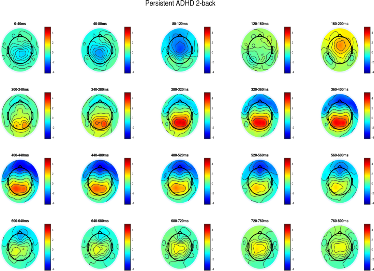


Figure S1. Bain electrical activity mapping of three groups during n-back tasks.


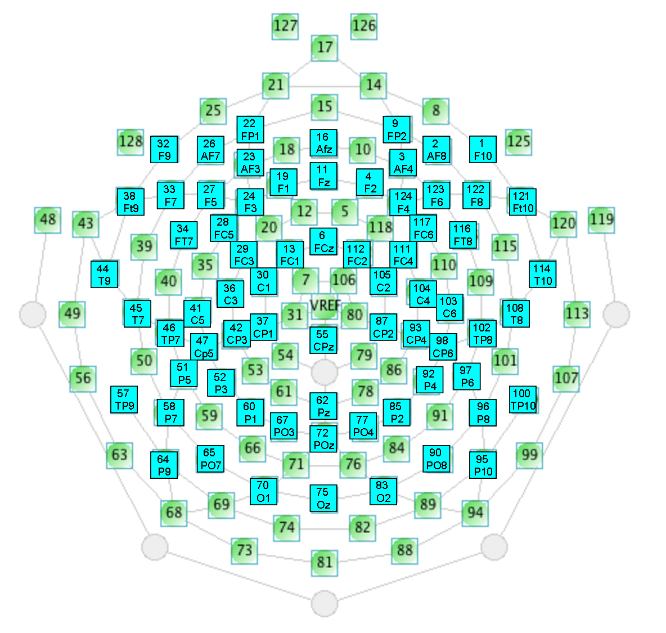


Figure S2. The 128-electrode location and selected electrode Fz and Pz.
